# Environmental health research needed to inform strategies, policies, and measures to manage the risks of anthropogenic climate change

**DOI:** 10.1186/s12940-021-00792-1

**Published:** 2021-10-01

**Authors:** Kristie L. Ebi

**Affiliations:** Center for Health and the Global Environment (CHanGE), University of Washington, Seattle, WA 98195 USA

**Keywords:** Human health, Climate change, Adaptation, Mitigation

## Abstract

Anthropogenic climate change is affecting population health and wellbeing worldwide. The two main policy options to prepare for and manage these risks are adaptation and mitigation; significantly increased investments in each are urgently needed. However, medical research councils worldwide have provided minuscule amounts of funding for environmental health research to provide timely and useful insights on effectively protecting vulnerable populations and regions, for building climate-resilient health systems, and for promoting health system-related greenhouse gas emission reductions in a changing climate.

Humans are at the core of anthropogenic climate change, from engaging in activities that emit greenhouse gases, to implementing adaptation and mitigation activities to avoid further risks, to experiencing the consequences for human health and well-being, particularly on the most vulnerable populations. However, anthropogenic climate change is not just a future problem. The need for urgent and immediate action was highlighted in a recent Editorial published simultaneously in numerous medical journals [1]. Climate change is a whole of society challenge that is already causing adverse injuries, illnesses, disabilities, and deaths, and affecting the functioning of healthcare infrastructure [2]. The consequences of climate change for health-determining sectors such as food, water, and urban, including the adaptation and mitigation actions implemented in those sectors, also will affect health and wellbeing.

The two primary policy responses to climate change are mitigation (reduction of greenhouse gas emissions) and adaptation (the process of adjustment to actual or expected climate and its effects, seeking to moderate or avoid harm or exploit beneficial opportunities). Focusing on mitigation to keep warming to 1.5–2.0 °C ignores the inherent inertia in the climate system that will continue to drive anthropogenic climate change for several decades [3] and what that increased warming could mean for the magnitude and burden of climate-sensitive health outcomes. A synthesis of projections for six climate-sensitive health outcomes concluded risks will continue to increase as the global mean surface temperature increases, with the pace of adaptation altering the extent of risk [4]. In other words, adaptation and mitigation are equally important.

Equal to the recent call for emergency action to limit global temperature increases [1] is the urgent need to invest in research to inform developing and implementing strategies, policies, and programs to proactively prepare for and manage increasing erratic weather patterns, including extreme weather and climate events;, increasing threats to water and nutrition safety and security;, changes in the geographic range of infectious diseases;, changes in the magnitude and pattern of respiratory diseases from reductions in air quality;, reductions in mental health and wellbeing;, and potential consequences of migration and conflict. Research needs to focus on protecting the health and wellbeing of vulnerable populations and communities (e.g., [5–8]) but should not delay the urgent and immediate actions needed today.

Focusing on mitigation implicitly assumes there is time to conduct basic research to understand how the magnitude and pattern of risks could change at some future point, and then to use the insights generated to design, implement, and evaluate adaptation options. Key research needs to inform effective risk management include quantifying exposure-response relationships for the full range of climate-sensitive health outcomes; projecting risks at spatial and temporal scales useful for decision-making, under a range of climate and development scenarios; developing best practices for designing, implementing, and evaluating health interventions that benefit the most vulnerable over spatial and temporal scales; understanding limits to adaptation and the associated loss and damage; and estimating the health co-benefits of mitigation policies and technologies. Research needs to be conducted in the context of other drivers of climate-sensitive health outcomes, such as population growth and ageing, urbanization, and socioeconomic change. The research focus should be broad, include emerging issues and ensure systems-based approaches are used to further understanding of the complex dynamics, including synergies and trade-offs, within health systems and across other sectors. Our journal continues to contribute evidence on all these aspects, noting that the impact of this evidence on policy decisions has been slow.

However, medical research councils worldwide have provided miniscule amounts of research funding, sending the message that climate change and health is not an issue worthy of investigation. Exceedingly low funding translates into a lack of career possibilities for researchers and practitioners, perpetuating a vicious cycle. In 2016, it was estimated that the U.S. National Institutes of Health (NIH) committed 0.025% of their annual research budget to climate change and health [9] and had been doing so for years. The situation was only marginally better in the European Union Horizon 2020 (0.04% of the budget) [9] and was equally bleak in Australia [10]. Under the Biden Administration, climate change and health research within the U.S. NIH would increase to 0.2% of a nearly USD 52 billion budget: both a significant increase and a tiny investment compared with the magnitude of the challenges.

It is long past time for medical research councils to fund the research and practice needed to protect population health and health systems in a changing climate, while promoting reductions in greenhouse gas emissions.

## Data Availability

Not applicable.
